# Application of Human Epineural Conduit Supported with Human Mesenchymal Stem Cells as a Novel Therapy for Enhancement of Nerve Gap Regeneration

**DOI:** 10.1007/s12015-021-10301-z

**Published:** 2021-11-17

**Authors:** Maria Siemionow, Marcin Michal Strojny, Katarzyna Kozlowska, Sonia Brodowska, Wiktoria Grau-Kazmierczak, Joanna Cwykiel

**Affiliations:** 1 Poznan University of Medical Sciences, Poznan, Poland; 2 Department of Orthopaedics, University of Illinois at Chicago, Chicago, IL USA

**Keywords:** Peripheral nerve repair, Human Epineural conduit, Mesenchymal stem cells, Regenerative medicine, Autograft, Nerve regeneration

## Abstract

Various therapeutic methods have been suggested to enhance nerve regeneration. In this study, we propose a novel approach for enhancement of nerve gap regeneration by applying human epineural conduit (hEC) supported with human mesenchymal stem cells (hMSC), as an alternative to autograft repair. Restoration of 20 mm sciatic nerve defect with hEC created from human sciatic nerve supported with hMSC was tested in 4 experimental groups (n = 6 each) in the athymic nude rat model **(**Crl:NIH-*Foxn1*^*rnu*^): 1 - No repair control, 2 - Autograft control, 3 - Matched diameter hEC filled with 1 mL saline, 4 - Matched diameter hEC supported with 3 × 10^6^ hMSC. Assessments included: functional tests: toe-spread and pinprick, regeneration assessment by immunofluorescence staining: HLA-1, HLA-DR, NGF, GFAP, Laminin B, S-100, VEGF, vWF and PKH26 labeling; histomorphometric analysis of myelin thickness, axonal density, fiber diameter and myelinated nerve fibers percentage; Gastrocnemius Muscle Index (GMI) and muscle fiber area ratio. Best sensory and motor function recovery, as well as GMI and muscle fiber area ratio, were observed in the autograft group, and were comparable to the hEC with hMSC group (*p* = 0.038). Significant improvements of myelin thickness (*p* = 0.003), fiber diameter (*p* = 0.0296), and percentage of myelinated fibers (*p* < 0.0001) were detected in hEC group supported with hMSC compared to hEC with saline controls. At 12-weeks after nerve gap repair, hEC combined with hMSC revealed increased expression of neurotrophic and proangiogenic factors, which corresponded with improvement of function comparable with the autograft control. Application of our novel hEC supported with hMSC provides a potential alternative to the autograft nerve repair.

**Graphical Abstract:**

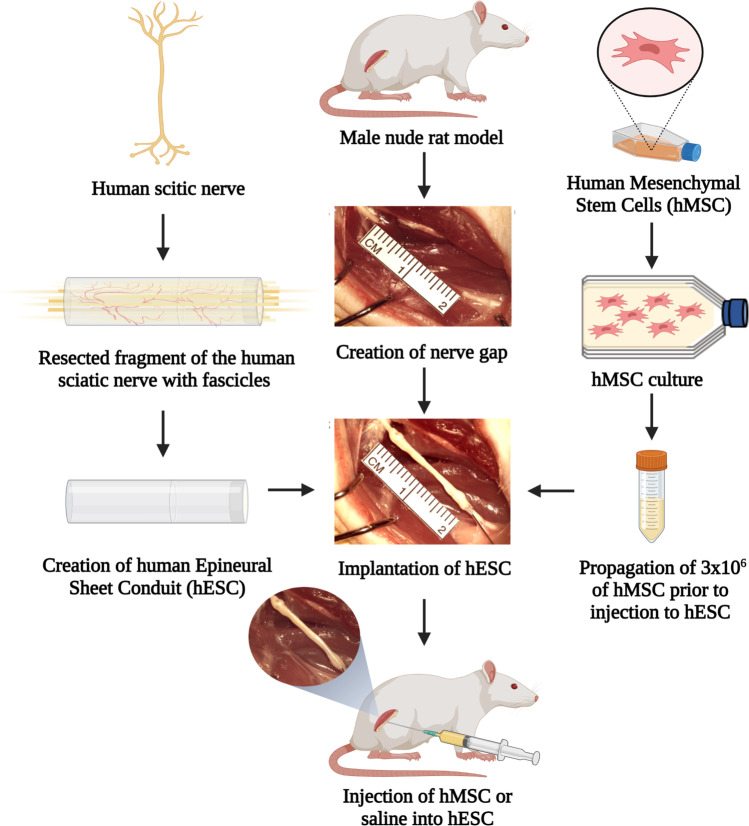

## Introduction

Peripheral nerve injuries, most commonly caused by traumatic events, result in severe motor disabilities in 2.8% of trauma patients, predominantly in the younger population [1, 2]. It is estimated that approximately 100,000 patients undergo peripheral nerve injury surgery every year in the United States and Europe [3, 4]. Stretch-related injuries are most prevalent among civilian patients’ population, closely followed by lacerations, accounting for about 30% of all cases [5]. The optimal surgical management in the repair of peripheral nerve injuries is tension-free repair [3]. End-to-end coaptation of nerve stumps performed under tension causes microvascular flow disruption within the nerve, resulting in ischemia, nerve fibrosis, and eventually poor recovery outcomes [6]. When a nerve gap resulting from nerve retraction, fibrosis, or tissue loss occurs, an alternative surgical strategy must be applied to repair the injured nerve. Available methods of nerve repair include peripheral nerve autograft or allograft, nerve transfer, end-to-side coaptation, and nerve conduits. Each method has its benefits and risk of complications, which must be considered while choosing the proper method of nerve repair. Despite early diagnosis and accurate nerve repair with modern surgical techniques, functional recovery never reaches pre-injury level due to factors that include but are not limited to the type and level of injury, the integrity of the surrounding tissues, timing of the surgery, and changes in spinal cord neurons and end organs [7–9].

The nerve conduits’ primary role is to provide axonal guidance, separate the developing axons from surrounding tissues and restrict inflammation as well as fibrous tissue ingrowth. Conduits, both biological and synthetic, have demonstrated varying success rates. They are extensively investigated to bridge the nerve gaps and include veins, arteries, tendons, epineurium, silicone and polyglactin mesh. In recent years, significant interest has been devoted to epineural sheath conduits. As a naturally occurring tissue surrounding the nerve, epineural sheath, which lacks Schwann cells, provides the surgeon with an ideal allogenic material that does not require immunosuppression [10, 11]. Siemionow’s Laboratory has extensively researched the use of human epineural conduit consisting of human epineural sheath for peripheral nerve gaps bridging [12–14]. In our previous studies, 20 mm sciatic nerve gaps were created in the rat model, followed by epineural tube repair with or without bone marrow stromal cells (BMSC). Findings demonstrated comparable results between the epineural tube/BMSC conduits and autograft repair [15].

Human mesenchymal stem cells (hMSC) are multipotent stem cells with the potential to differentiate into mesodermal and other embryonic lineages depending on the signal from their microenvironment. Mesenchymal stem cells (MSC) can be isolated from the bone marrow, adipose tissue, amniotic fluid, endometrium, dental tissues, and umbilical cord. Their potential to differentiate into neural cells provides promise for future application of cell-based therapies for many neurological disorders [16–18]. In addition, neuroprotective and neurorestorative properties of cell-based therapies were tested in different animal models and clinical trials including neurodegenerative diseases such as Parkinson’s and Huntington’s disease, as well as traumatic brain injuries and strokes [19]. MSC’s are weakly immunogenic and improve neuronal function by secreting neurotrophic, growth factors, and cytokines such as brain-derived neurotrophic factor (BDNF), ciliary neurotrophic factor (CNTF), glial cell line-derived neurotrophic factor (GDNF), insulin-like growth factor (IGF), interleukin 6 (Il6) and nerve growth factor (NGF), that promote differentiation, proliferation and survival of nerve cells [20–25]. Moreover, it is reported that MSCs produce a variety of angiogenic cytokines, such as vascular endothelial growth factor (VEGF), basic fibroblast growth factor (FGF), hepatocyte growth factor (HGF), insulin-like growth factor 1 (IGF-1), monocyte chemoattractant protein (MCP)-2, and MCP-3. The above-mentioned properties of the human mesenchymal stem cells make them a promising option as the supportive therapy for enhancement of peripheral nerve regeneration.

We hypothesize that our novel approach for enhancement of nerve regeneration, combining the hEC supported with hMSC will result in optimal recovery of the damaged peripheral nerves. Furthermore, we expect that this new method of peripheral nerve gap repair will result in similar if not better outcomes when compared to the autograft, which is currently considered the method of choice in peripheral nerve gap repair [26, 27].

## Materials and Methods

### Experimental Animals

Animal care and experimental protocols were approved by the Institutional Animal Care and Use Committee (IACUC) of University of Illinois at Chicago, which is approved by the American Association for the Accreditation of Laboratory Animal Care (AAALAC). All animals received humane care in compliance with the ‘Principles of Laboratory Animal Care’ formulated by the National Society for Medical Research and the ‘Guide for the Care and Use of Laboratory Animal Resources. In this experimental study, we used a total of 24 male athymic homozygous nude rats **(**Crl:NIH-*Foxn1*^*rnu*^*, Charles River Laboratories, USA)* weighing between 150 and 250 g. Animals were housed in pairs in hooded cages at room temperature, on a light-dark schedule of 14/10, with no limitation of food or water.

### Human Mesenchymal Stem Cells Culture, Viability and Labeling

Mesenchymal stem cells (MSC, Lonza, Inc., Switzerland) were cultured in MSC growth media supplemented with MSC growth supplement, L-glutamine, and Gentamicin-Amphotericin-B (Lonza, Inc., Switzerland) as previously reported [28]. Cultured MSC (60–70% confluence) were harvested between passages 5–8 using a standard procedure with 0.25% trypsin-EDTA (Gibco-Thermo Fischer, USA) [29]. Prior to injection, MSC were labeled using PKH26 fluorescent dye (Sigma-Aldrich, UK) according to the manufacturer’s instructions and as reported previously [28]. The efficacy of MSC labeling with PKH26 fluorescent dye was confirmed prior to cell delivery by flow cytometry and confocal microscopy. Briefly, unstained MSC controls and PKH26 labeled MSC were fixed in 4% paraformaldehyde and analyzed using a Fortessa flow cytometer (Becton Dickinson, USA). For confocal microscopy, cells were spun onto positively charged lysine-coated slides and counterstained with DAPI (Vector Laboratories, USA). Next, slides were examined using Zeiss Meta confocal microscope and analyzed with ZEN software (Zeiss, Germany). The viability and number of hMSC before and after PKH26 labeling were tested using 0.4% Trypan Blue.

### Assessment of Mesenchymal Stem Cells Phenotype

The phenotype of MSC was confirmed prior to cell delivery by flow cytometry. Cells suspended in staining buffer containing 1% BSA and 0.05% sodium azide in D-PBS were incubated with Rat BD Fc Block (BD Biosciences, USA) for 5 min., and later with fluorochrome-conjugated antibodies: APC anti-human CD29, FITC anti-human CD44, BV421 anti-human CD90, APC-anti-human CD105, BV421 anti-human CD73 (BD Biosciences, USA), BV570 anti-human CD45 (Biolegend, USA), APC mouse anti-human CD34, APC mouse anti-human CD14 (BD Biosciences) for 40 min. Following washing with a cell staining buffer, cells were fixed with 1% neutral buffered formalin overnight. Samples resuspended in 1% BSA were assessed on the following day using a BD LSR II cell analyzer (Becton Dickinson, USA)*.*

### Human Epineural Conduit

**Fig. 1:**
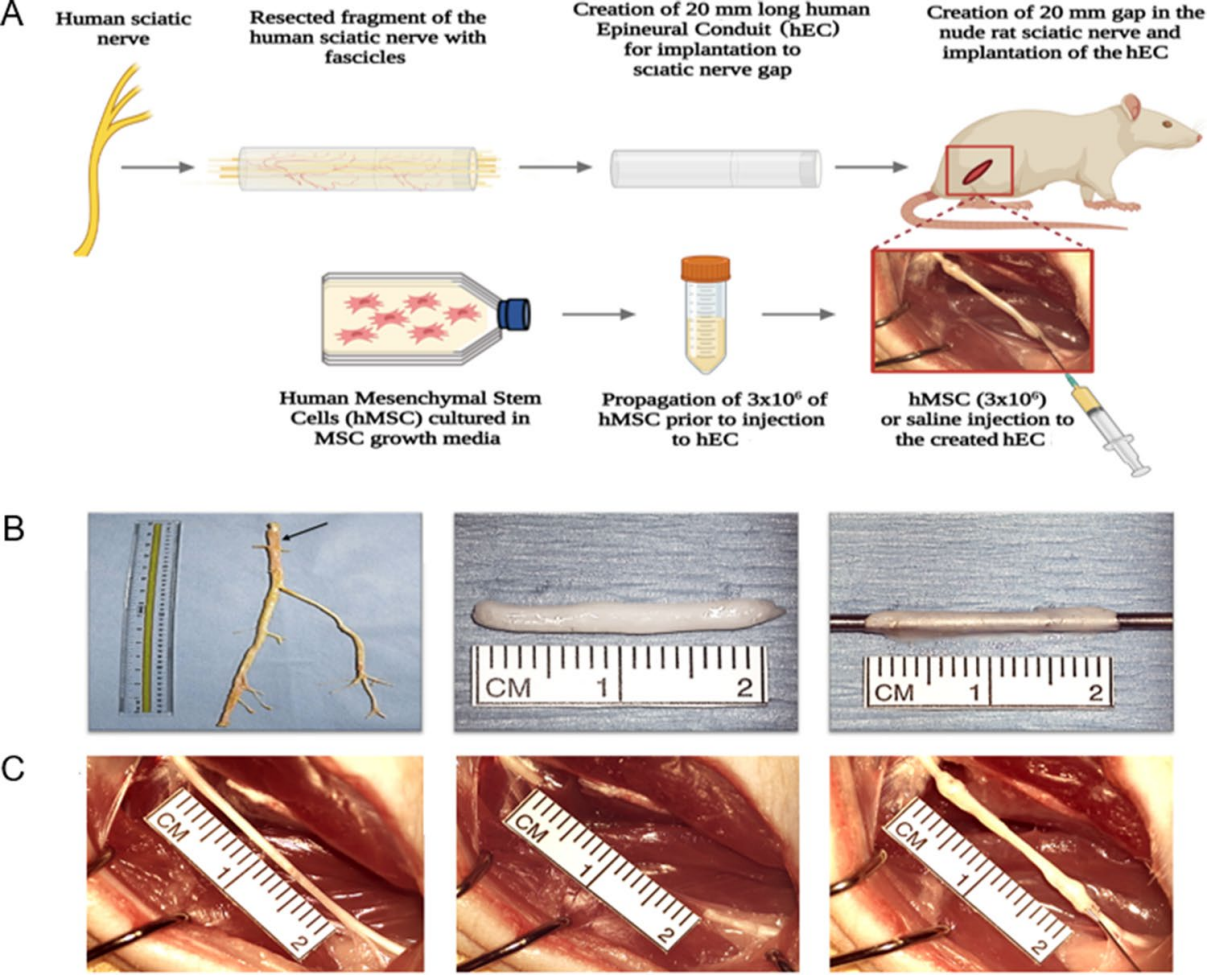
The outline of experimental study design for creation and application of the human epineural conduit (hEC) as a novel therapy for enhancement of nerve gap regeneration. **A** Schematic representation of the creation and application of the human epineural conduit (hEC) supported with human mesenchymal stem cells (hMSC). **B** Creation of the human epineural conduit (hEC) from the sciatic nerve. Human sciatic nerve with branches purchased from the Musculoskeletal Transplant Foundation. The arrow marks the human sciatic nerve (left picture). Epineural sheath after removal of the fascicles during harvesting (middle picture). Empty human epineural conduit, ready for implantation to fill the sciatic nerve gap (right picture). **C** Implantation of human epineural conduit into the sciatic nerve gap. 20 mm long segment of the rat sciatic nerve before resection (left picture). Creation of a 20 mm gap in the rat sciatic nerve (middle picture). Implantation of hEC into the 20 mm gap followed by injection of either hMSC or saline into the conduit (right picture)

Frozen human sciatic nerves were purchased from the Musculoskeletal Transplant Foundation (NJ, USA) in sterile conditions and on dry ice. The nerve was defrosted by placing it onto a warm water circulating heating pad (T/Pump, Gaymar Industries, USA) at 38 °C. For experimental groups, nerve epineural conduits were prepared in aseptic conditions before the nerve repair procedure. The 3–4 cm long section of the sciatic nerve without side branches was resected from the nerve. Using microsurgical tools under a surgical microscope (Wild 691, Leica Microsystems, Germany), the epineurium was separated from the fascicles using a method well established by our laboratory [15, 30, 31]. The conduit was thoroughly inspected for any tears or damage, which would preclude it from implantation. Next, the conduit was cut into 2 cm long pieces and placed in saline before implantation into the sciatic nerve gap. Figure 1A-C presents an outline of: the study design (A), technique of the hEC creation (B) and technique of hEC implantation into the sciatic nerve gap (C).

### Surgical Procedure

Each rat was weighed before surgery using a triple beam scale (700/800 series, OHAUS®, USA). For all the surgical procedures, Isoflurane (Terrell Isoflurane, Piramal Critical Care Inc., USA) inhalation (induction 5% until unconscious, maintenance 1.5–2.5%) was used through the SurgiVet Vaporizer (Smiths Medical, USA). Pain control was achieved with a subcutaneous injection of Buprenorphine SR (1.2 mg/kg) 15 min before the first skin incision. After the animal was anesthetized, the rat was placed on the left side, the right hind limb was shaved, and a thin coat of hair remover lotion (Nair, Church & Dwight Co., USA) was applied for 3–5 min and then wiped with gauze. The animal was placed on the surgical table, and the surgical site was cleansed with a 5% povidone-iodine solution (Betadine, Purdue Products L.P., USA). Surgery was performed at room temperature, and animals were placed on a warm water circulating heating pad (T/Pump, Gaymar Industries, USA) before and during recovery time after surgery. A 3 cm oblique surgical incision was performed at the right gluteal area and the gluteus superficialis and biceps femoris muscles were visualized. Following muscle dissection, the right sciatic nerve was exposed (from the sciatic notch to the bifurcation into terminal branches), and an intact 20 mm segment of the sciatic nerve was resected (Fig. 1C).

Following excision, the surgical wound of the control group without nerve repair was closed. In the autograft control and conduit groups, on the proximal and distal stumps of the sciatic nerve, eight epineural sutures (10–0 Vicryl) were placed and passed through the proximal and distal edge of the conduit, respectively. After completion of nerve gap repair with hEC, the conduits were filled with either saline or hMSC. Next, the gluteus superficialis and biceps femoris muscles were approximated using a 4–0 interrupted vicryl suture. The skin was approximated using interrupted 5–0 monocryl sutures (Ethicon, USA) and an antibiotic cream (Neosporin, Johnson & Johnson, USA) was applied. The surgery was performed using the aseptic technique by one surgeon using the microsurgical operating microscope (Wild 691, Leica Microsystems, Germany) under 20x-40x magnification. Animals were monitored 24 h post-surgery.

### Experimental Groups

Twenty four nude rats (Crl:NIH-*Foxn1*^*rnu*^) were investigated in four experimental groups of 6 rats each. All animals were allocated to each experimental group in random order. Group 1 served as the no repair control. Following resection of 20 mm of a sciatic nerve segment, no further surgical intervention was applied. In Group 2, repair of the nerve defect was made using the 20 mm segment of the nerve autograft. In Group 3 - sciatic nerve excision was followed by human epineural conduit application between proximal and distal nerve stumps and was next filled with 1 mL of saline solution. In Group 4, after bridging the 20 mm nerve gap with human epineural conduit, the tube was filled with 3 × 10^6^ hMSC suspended in 1 mL of saline under 20X surgical microscope magnification. Representative pictures of implantation of the human epineural conduit are presented in Fig. 1C. Functional assessments were performed at 1, 3, 6, 9, and 12-weeks after nerve gap repair.

### Postsurgical Supportive Treatment

For the first 24 h, each rat was individually quarantined with a collar around the rats’ neck for protection against wound biting. The next day the collar was taken off, and the rat was placed into the original cage. Postoperative pain control was provided with Buprenorphine (0.1 mg/kg) twice a day for the first two days. Animals were physically examined daily for the first 14 days post-surgery to assess the wound site. Signs of morbidity, lack of eating or drinking, weight loss, inability to locomotor activities, symptoms of pain or distress such as rough hair or hunched posture were taken into account. The animals were also under the University of Illinois at Chicago veterinary team’s care, which examined each rat once a week.

### Assessment Methods

The animals were evaluated for functional recovery at 1, 3, 6, 9 and 12-weeks after nerve gap repair. All animals were euthanized at 12-weeks post-surgery using euthanasia solution SomnaSol (Henry Schein, Inc., USA). Both gastrocnemius muscles and the sciatic nerve repair segments were harvested for histological and immunological examination.

#### Assessment of Muscle Denervation Atrophy

Gastrocnemius muscle index (GMI) was measured to evaluate the muscle denervation atrophy. The muscle was excised from both limbs at the study endpoint of 12-weeks after nerve defect repair. Gastrocnemius muscle (GM) weight was measured immediately using a digital scale (Ohaus Precision Standard, Germany). The wet weight of the ipsilateral GM was related to the contralateral gastrocnemius, and the GMI was calculated. The percentage value of the GMI index represented recovery of the denervation atrophy of the gastrocnemius muscle on the operated side, with a 100% GMI indicating full recovery.

#### Histomorphometric Analysis

After GMI was assessed, the gastrocnemius muscles were fixed in formalin. Routine H&E-stained paraffin sections were prepared after the cross-sections of the muscle samples were taken. Six non-overlapping fields were chosen from each muscle sample, with three hundred muscle fibers assessed in total. Images were taken using a Leica DM4000B Compound Microscope (Leica Microsystems, Germany) with a Qimaging Retiga 2000R Color Digital Camera (Teledyne Photometrics, USA), then digitized and assessed using Image-Pro Plus, Ver 6.3.0.512 (Media Cybernetics, USA). The average muscle fiber areas were compared between the right and left limb in each animal, and the values were expressed as the R/L ratio.

#### Functional Motor Assessment

The toe-spread test was used for the evaluation of motor recovery. In an uninjured limb, the rat extends and abducts the hindfoot toes when the rat is held up by the tail. The toe-spread test was graded between 0 and 3 in the following manner: no movement = grade 0, any sign of movement of the toes = grade 1, abduction of the toes = grade 2, abduction and extension of toes = grade 3.

#### Functional Sensory Assessment

The pinprick test was used for the evaluation of sensory recovery. Using Adson’s toothed forceps, pressure was applied to the skin of the right hind limb of the rat. This was performed starting from the toe to the level of the knee joint until a retraction of the limb and/or a vocal response from the painful stimulus was obtained. Attention was paid not to pinch the deep tissues and periosteum of the limb. Stimulus-response was graded between 0 and 3 in the following manner: no sensation was elicited on the limb = grade 0, withdrawal response between the knee and ankle = grade 1, withdrawal response between the ankle and toes = grade 2, and withdrawal response to the pinch of the toes = grade 3. The test was performed at least three times at each evaluation stage to prevent incidental false positive results.

#### Macroscopic Evaluation of the hEC

After the rat’s euthanasia, a 3 cm incision was made in the gluteal region of the right hind limb to visualize the right sciatic nerve, as described earlier. Once the nerve and the graft were visualized, the following assessments were performed: adhesions with surrounding tissues or local signs of inflammation, structure, shape, and integrity of the graft, fascicle-like structures inside the conduit, presence of atrophy signs of the nerve distally to the conduit and assessment of vascularization of the graft.

#### Immunostaining

Expression of growth factors involved in nerve regeneration was assessed by monoclonal antibodies and immunofluorescence techniques. Freshly dissected nerve conduit from both the proximal and distal stump was suspended and snap-frozen in O.C.T. compound. Tissue slides were cut for 1 μm slides and fixed for 10 min in acetone. Next, the sections were rinsed in Tris Buffered Saline (TBS, Agilent Technologies, Inc., USA) and incubated with mouse antirat vWF, VEGF (Thermo Fischer Scientific, USA) and S-100 (Abcam, Inc., UK), rabbit antirat GFAP (Thermo Fisher Scientific, USA), Laminin B and NGF (Abcam, Inc., UK), and mouse anti-human HLA-1 and HLA-DR (Abcam, Inc., UK) monoclonal antibody for 30 min. Incubation using secondary antibodies was performed using goat anti-mouse or goat anti-rabbit IgG Cross-Absorbed Alexa Fluor 488 (Thermo Fischer Scientific, USA). PKH26 staining of hMSC prior to implantation assessed the presence of hMSC in the conduit. The slides were stained with DAPI and analyzed using a Leica DM 4000B Compound Microscope (Leica Microsystems, Germany) with a Qimaging Retiga 2000R Color Digital Camera (Teledyne Photometrics, USA) and digitalized and assessed using Image-Pro Plus, Ver 6.3.0.512 (Media Cybernetics, USA). Assessment of immunoreactivity was scored as follows: 0 = no staining; 1 = weak; 2 = moderate; and 3 = strong.

#### Toluidine Blue Staining

Tissue samples of the conduit from both the proximal and distal stump of the nerve were excised, immersed, and fixed in 4% glutaraldehyde. The specimens were post-fixed using 4% aqueous osmium tetroxide and embedded in Araldite 502 following the manufacturer’s instructions. Toluidine blue stain was used to stain 1 μm thick cross-sections for light microscope evaluation of histological samples. Six non-overlapping fields were chosen from each nerve. Images of these nerves were taken using a Leica DM 5500B Compound Microscope with a Leica DFC290 Color Digital Camera (Leica Microsystems, Germany), digitalized, and evaluated using Image-Pro Plus, Ver 6.3.0.512 (Media Cybernetics, USA). Each image was assessed for myelin thickness, axonal density, fiber diameter, and percentage of the myelinated nerve fibers.

## Statistical Analysis

Data are expressed as mean ± SEM (standard error of the mean). GraphPad Prism (ver. 9.2.1) software was used to perform statistical analysis. One-way or two-way ANOVA with post-hoc Tukey’s test were used for group comparisons to define statistical significance. Results were considered statistically significant when *p* < 0.05.

## Results

### Confirmation of hMSC Phenotype, Viability and PKH26 Labeling Prior to MSC Injection into the hEC

**Fig. 2:**
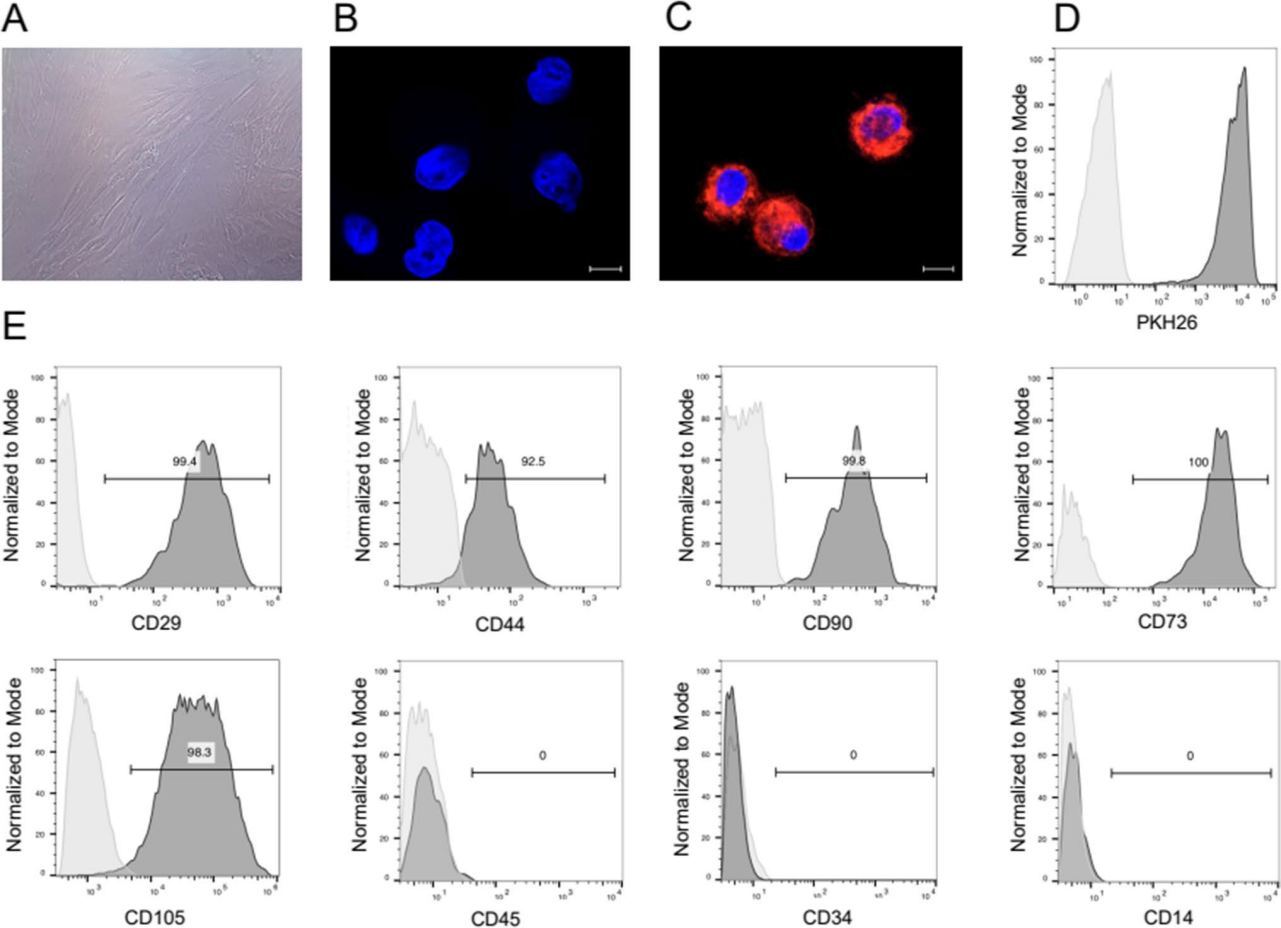
Phenotype characterization of the human mesenchymal stem cells (hMSC) for the application as a supportive therapy for human epineural conduit (hEC). **A** “Fibroblast- like” morphology of hMSC in cell culture after 8 passages. **B, C** Representative images confirming the efficacy of PKH26 hMSC labelling via confocal microscopy: (**B**) Unlabeled hMSC, (**C**) PKH26 labelled hMSC; for merging: Blue-DAPI, Red-PKH26, scale 10 μm; (**D**) Flow cytometry histogram confirming the efficacy of PKH26 hMSC labelling: unstained hMSC (light grey histogram on the left) superimposed on the PKH26 labeled hMSC (dark grey histogram on the right). **E** Flow cytometry evaluation of hMSC phenotype. The representative histograms confirm the presence of CD29, CD44, CD90, CD105, CD73 positive cells and lack of expression of hematopoietic markers: CD45, CD34 and CD14

As anticipated, in vitro cultured hMSC presented plastic adherence and “fibroblast-like” morphology (Fig. 2A). Confocal microscopy and flow cytometry confirmed that PKH26 is an efficient dye for hMSC labeling with low cell toxicity [15]. The analysis showed strong fluorescent labeling of hMSC (Fig. 2B-C). The hMSC viability ranged between 80 and 90%, as tested by Trypan blue staining. The characterization of hMSC phenotype performed using flow cytometry confirmed strong expression of hMSC specific cell surface markers, including CD29, CD44, CD90, CD73, CD105, as well as lack of expression of hematopoietic markers, such as CD45, CD34, and CD14 [32] (Fig. 2D-E).

### Confirmation of Lack of Inflammation, Adhesions, or Fibrosis at 12-Weeks after hEC Implantation

At 12-weeks after implantation, each hEC conduit was evaluated macroscopically before harvesting for histological and immunofluorescence assessments. No adhesions or local signs of inflammation around the conduits were found. Each conduit had a well-preserved structure, shape, and integrity with good vascularization of the graft. We confirmed the presence of fascicle-like structures inside the grafts. No signs of atrophy of the nerve distally to the conduit were observed.

### Confirmation of Improvement of Gastrocnemius Muscle Morphology and Regenerative Effect of hEC Supported with hMSC at 12-Weeks after Sciatic Nerve Gap Repair

To assess the effect of sciatic nerve gap repair with hEC on gastrocnemius muscle regeneration, Gastrocnemius Muscle Index and Muscle Fiber Area Ratio were measured.

#### Gastrocnemius Muscle Index

**Fig. 3:**
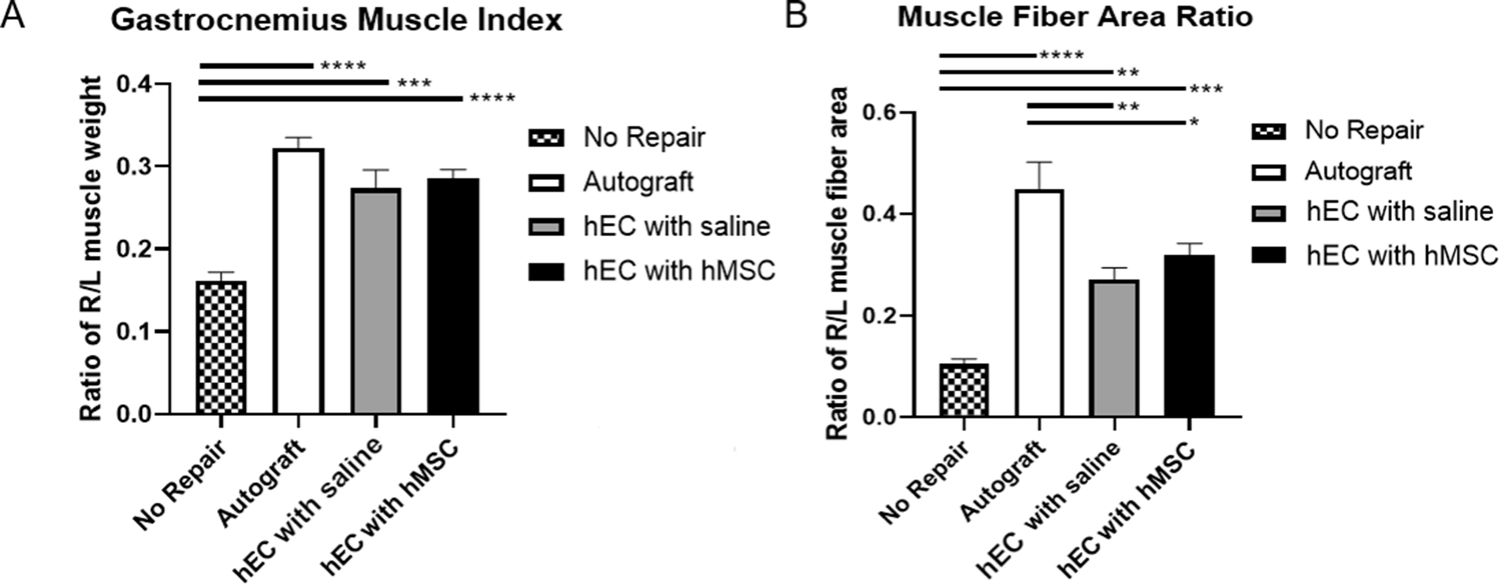
Assessment of muscle denervation atrophy by Gastrocnemius Muscle Index (GMI) and muscle fiber area ratio at 12-weeks follow-up after sciatic nerve repair with the hEC. **A** GMI was significantly lower in the no repair group when compared to the autograft group, the hEC with hMSC group and the hEC with saline group. **B** Significant differences in the muscle fiber area ratio were revealed between the no repair group when compared to the autograft, the hEC with MSC and the hEC with the saline group. Differences were also statistically significant between the autograft when compared to the hEC with hMSC and the hEC with saline group. The graphs represent mean values with SEM, statistical significance is marked with asterisks: *p < 0.05, **p < 0.01, ***p < 0.001, ****p < 0.0001

Gastrocnemius muscle reinnervation, measured by GMI, showed significant differences between no repair (0.162 ± 0.01) when compared to the autograft group (0.323 ± 0.012; *p* < 0.0001), the hEC with hMSC group (0.285 ± 0.011; *p* < 0.0001) and the hEC with saline group (0.274 ± 0.022; *p* = 0.0001) at 12-weeks after nerve gap repair (Fig. 3A).

#### Muscle Fiber Area Ratio

Muscle fiber area ratio after 12-weeks follow-up in 4 groups (no repair, autograft, hEC with saline, and hEC with hMSC) is depicted in Fig. 3B. As expected, the best results were obtained for the autograft group (0.45 ± 0.053). Significant changes were revealed between no repair group (0.11 ± 0.01) when compared to the autograft (0.45 ± 0.05; *p* < 0.0001), the hEC with hMSC (0.32 ± 0.02; *p* = 0.0005) and the hEC with saline group (0.27 ± 0.02; *p* = 0.0062). Differences were also statistically significant between the autograft (0.45 ± 0.05), when compared to the hEC with hMSC (0.32 ± 0.02; *p* = 0.038), and the hEC with saline group (0.271 ± 0.02; *p* = 0.0034).

### Confirmation of Improvement of Muscle Function at 12-Weeks after Sciatic Nerve Gap Repair with hEC Supported with hMSC

Standard functional tests of Toe-Spread Test and Pinprick Test were used to assess muscle function at 12-weeks after sciatic nerve repair with hEC supported with hMSC.

#### Functional Motor Assessment: The Toe-Spread Test

**Fig. 4:**
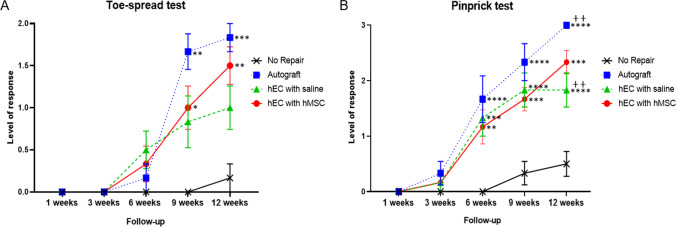
Functional assessment of nerve regeneration by toe-spread and pinprick tests up to 12-weeks after sciatic nerve repair with hEC. **A** Improvement of motor function assessed by toe-spread test began at week 6th in both conduit groups and the autograft. Significant difference was observed between the no repair and the autograft as well as the no repair group and the hEC with hMSC groups at 9-weeks. Similar trend was observed at 12-weeks after nerve repair between the no repair group and the autograft group as well as the no repair and the hEC with hMSC group. **B** Sensory function assessed by a pinprick test revealed significant difference between the no repair group when compared to the autograft and both, the hEC with saline and the hEC with hMSC at 6-weeks. At 9-weeks and 12-weeks follow-up the same trend was observed. Moreover, at 12-weeks after nerve repair, the statistical significance was observed between the autograft and the hEC with saline group. The graphs represent mean values with SEM, statistical significance is marked with asterisks: *p < 0.05, **p < 0.01, ***p < 0.001, ****p < 0.0001 and with crosses ++ *p* < 0.01

At 3-weeks follow-up, no return of the motor function was observed in all experimental groups. Results improved for the autograft and two of the experimental groups, with no significant differences observed between groups, at the 6-weeks follow-up. Significant results were noted at 9-weeks follow-up between the autograft (1.7 ± 0.2) and no repair group (0.0 ± 0.0; *p* < 0.01), as well as no repair (0.0 ± 0.0) and the hEC with hMSC group (1.0 ± 0.3; *p* < 0.05). At 12-weeks, the highest value was observed in the autograft group (1.8 ± 0.2), followed by the hEC with hMSC group (1.5 ± 0.2), both of which demonstrated a better motor function recovery, when compared to the hEC with saline group (1.0 ± 0.3); however, no statistically significant difference was observed between these groups. Significant difference was observed between no repair (0.2 ± 0.2) and the autograft (1.8 ± 0.2; *p* < 0.001) as well as no repair group (0.2 ± 0.2) and the hEC with hMSC (1.5 ± 0.2; *p* < 0.01) groups. Detailed results are presented in Fig. 4A.

#### Functional Sensory Assessment: The Pinprick Test

There were no significant changes observed between control and experimental groups up to 3-weeks after the nerve defect repair. The sensory function was recovering steadily from 6 to 12-weeks after gap repair in the autograft, the hEC with hMSC and the hEC with saline groups. At 6-weeks, there was a significant improvement in pinprick response between no repair and the autograft (0.00 ± 0.00 vs. 1.67 ± 0.42; *p* < 0.0001), no repair, when compared to the hEC with hMSC group (1.17 ± 0.3; *p* = 0.0017), as well as between no repair and the hEC with saline group (1.33 ± 0.33; *p* = 0.0002). At 9 weeks, the same trend was observed. Significant difference was revealed between no repair (0.33 ± 0.21) and the autograft (2.33 ± 0.33; *p* < 0.0001), no repair (0.33 ± 0.21) and the hEC with saline (1.84 ± 0.31; *p* < 0.0001) group as well as between no repair (0.33 ± 0.21) and the hEC with hMSC group (1.67 ± 0.21; *p* = 0.0002). At the 12-weeks endpoint, the pinprick test showed significantly improved sensory recovery in the autograft (3.0 ± 0.00; *p* < 0.0001), the hEC supported with hMSC (2.33 ± 0.21; *p* < 0.0001) and the hEC with saline groups (1.83 ± 0.31; *p* = 0.0002) when compared to no repair group (0.5 ± 0.22). Significant recovery was also detected between the autograft when compared to the hEC with saline group (3.0 ± 0.00 vs. 1.83 ± 0.31; *p* = 0.0017, Fig. 4B). There was no statistically significant difference in the sensory recovery between the autograft and the hEC supported with hMSC.

### Confirmation of Human Origin of the hEC at 12-Weeks after Sciatic Nerve Gap Repair

Presence of hMSC was confirmed by PKH26 staining and human origin of hEC by assessment of HLA-1 and HLA -DR expression in the hEC supported with hMSC.

#### PKH26

**Fig. 5:**
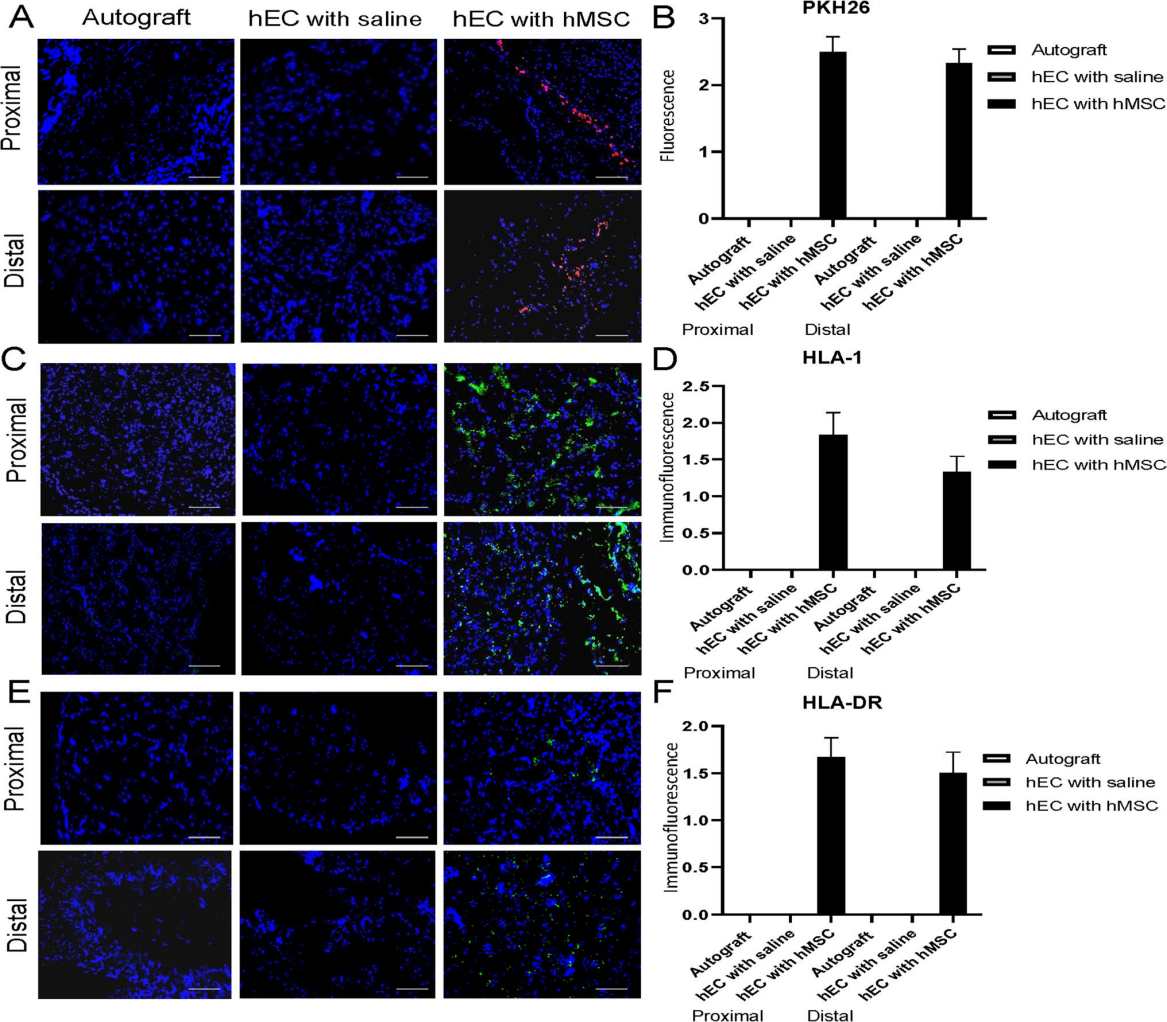
Presence of PKH26 labeled cells and expression of HLA-1 and HLA-DR within the proximal and distal end of the conduit assessed by fluorescence and immunofluorescence staining at 12-weeks after nerve repair with the hEC. **A, B** PKH26 labeled cells were detectable at the proximal and distal ends of the hEC supported with hMSC group. **C, D** HLA-1 expression was observed only in the hEC supported with hMSC group, confirming stem cell presence in the conduits. **E, F** The presence of HLA-DR was observed only in the hEC supported with hMSC group. Magnification 200x, scale bar 20 μm. The graphs represent mean values with SEM, statistical significance is marked with asterisks: *p < 0.05, **p < 0.01, ***p < 0.001, ****p < 0.0001

The presence of the PKH26 labeled cells was detected in the proximal and distal segments of the conduit in the hEC group supported with hMSC. The intensity of PKH26 labelling was higher at the proximal end, when compared to the distal end of the conduit. No PKH26 labeled cells were present in the hEC with the saline and the autograft group (Fig. 5A-B).

#### HLA-1

HLA-1 expression was confirmed only in the hEC with the hMSC experimental group. HLA-1 expression was higher at the proximal end of the conduit when compared to the distal end. Both the hEC with saline and the autograft groups did not express HLA-1 (Fig. 5C-D).

#### HLA-DR

HLA-DR expression was confirmed in the hEC with hMSC group. No expression was observed in the hEC with saline or the autograft group. There was a higher number of cells presenting HLA-DR at the proximal end, when compared to the distal segment of the conduit (Fig. 5E-F).

### Confirmation of Neuroregenerative Potential of hEC Supported with hMSC at 12-Weeks after Nerve Gap Repair

Neuroregenerative potential of hEC was evaluated by assessment of growth factor expression including: GFAP, S-100, Laminin B, NGF, vWF and VEGF.

#### GFAP

**Fig. 6:**
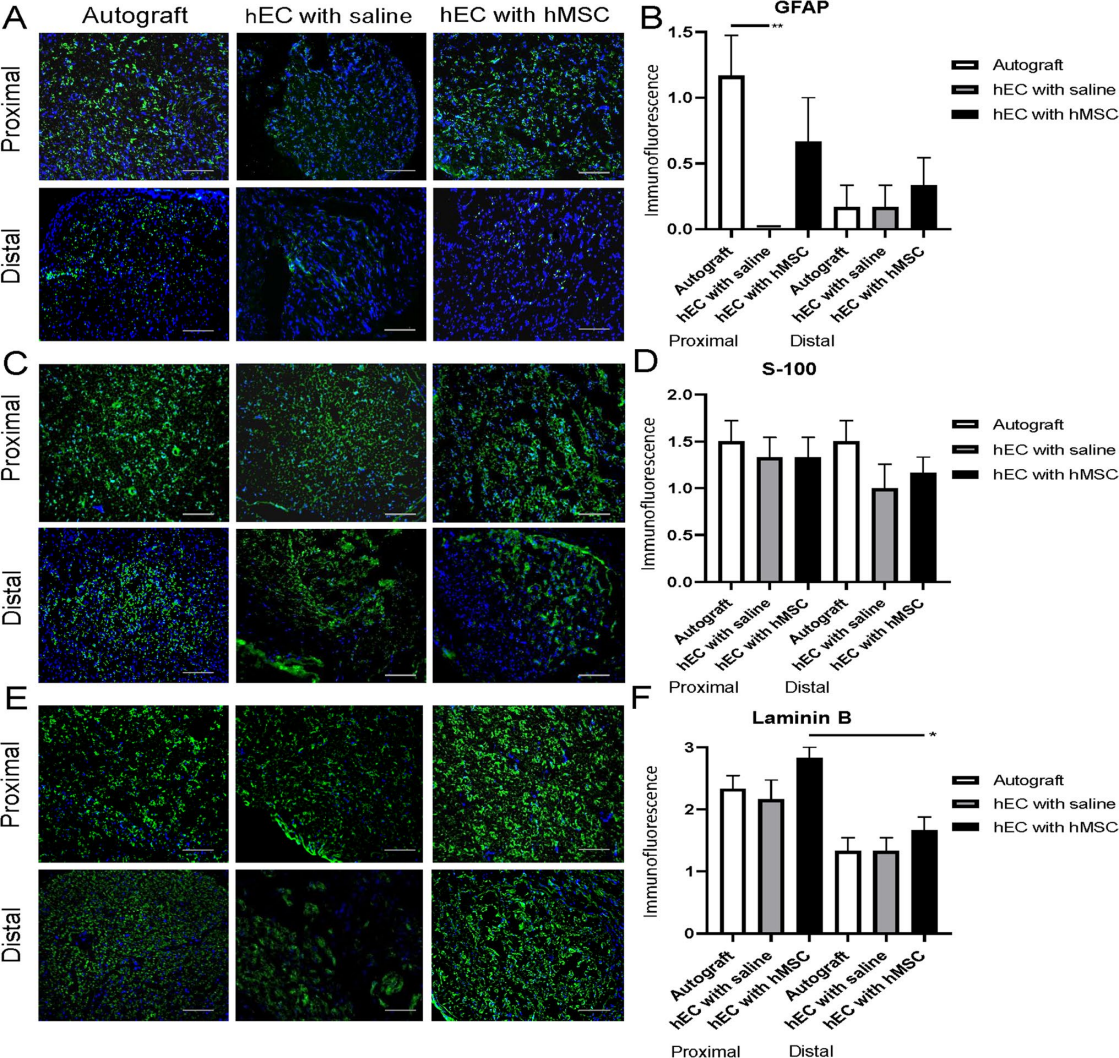
Expression of GFAP, S-100 and Laminin B within proximal and distal ends of the conduit in autograft, hEC with saline and hEC with hMSC groups assessed by immunofluorescent staining at 12-weeks after nerve repair with the hEC. **A, B** GFAP expression was the highest in the autograft group and moderate in the hEC with hMSC group at the proximal end, while in the hEC with saline group expression of GFAP was weak. **C, D** S-100 expression in the hEC group supported with hMSC was comparable to the autograft control group at both - proximal and distal ends of the conduit. In the hEC group filled with saline, S-100 expression was weak at both – proximal and distal ends of the conduit. **(E, F)** The highest level of Laminin B expression was detected at the proximal and distal end of the conduit in the hEC with hMSC when compared with the hEC with saline and the autograft group. Magnification 200x, scale bar 20 μm. The graphs represent mean values with SEM, statistical significance is marked with asterisks: *p < 0.05, **p < 0.01, ***p < 0.001, ****p < 0.0001

Expression of GFAP remained weak in the hEC with saline group at proximal and distal ends of the conduit. The highest expression of GFAP was revealed in the autograft group, however no significance was observed when comparing the hEC with hMSC group. A significant difference in the GFAP expression was detected at the proximal end of conduit between the autograft and the hEC with saline group (1.17 ± 0.31 vs. 0.00 ± 0.00; *p* = 0.017, Fig. 6A-B). At the distal end of the conduit the expression of GFAP was higher in the hEC supported with hMSC when compared to the autograft, but there was no significant difference detected.

#### S-100

Expression of Schwann cells marker S-100 was confirmed in all experimental groups with an increased level of expression present at the proximal ends of the conduits. No significant differences were found between the hEC with saline, the hEC with hMSC, and the autograft groups (Fig. 6C-D).

#### Laminin B

Expression of Laminin B, an axonal growth marker, was up-regulated at the proximal nerve end when compared to the distal end in the autograft, the hEC with hMSC and the hEC with saline groups with the significant difference between proximal and distal end of the conduit in the hEC with hMSC group (1.33 ± 0.21 vs. 1.00 ± 0.00, *p* = 0.0104). At the proximal end the expression of Laminin B reached higher value in hEC supported with hMSC when compared to the autograft group and the hEC with saline. The same trend was observed at the distal end of the conduit. No significant differences in Laminin B expression were detected between experimental and control groups (Fig. 6E-F).

#### NGF

**Fig. 7:**
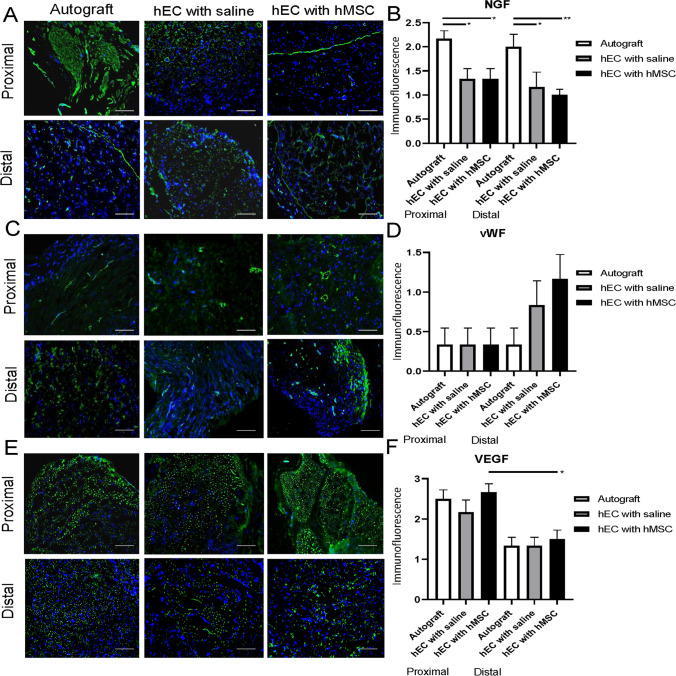
Expression of NGF, vWF and VEGF within proximal and distal ends of the conduit in the autograft, hEC with saline and hEC with hMSC groups assessed by immunofluorescent staining at 12-weeks after nerve repair with hEC. **A, B** Strong expression of NGF was noted in the autograft group at proximal and distal conduit end. Moderate expression was observed at both ends of the conduit in the hEC supported with hMSC and the hEC with the saline group. **C, D** Expression of vWF was weak in all groups at the proximal end. At the distal conduit end vWF expression was the highest in the hEC group supported with hMSC. Moderate level of vWF expression was detected in the hEC with saline group, whereas in the autograft group expression of vWF was the weakest. **E, F** The highest VEGF expression level was revealed in the hEC with hMSC group within proximal and distal conduit end. Magnification 200x, scale bar 20 μm. The graphs represent mean values with SEM, statistical significance is marked with asterisks: *p < 0.05, **p < 0.01, ***p < 0.001, ****p < 0.0001

The autograft group presented strong expression of NGF at the proximal and distal nerve ends. Significant changes were found in the autograft (2.17 ± 0.17) when compared to the hEC with hMSC (1.33 ± 0.21, *p* = 0.024) and the hEC with saline groups (1.33 ± 0.21, *p* = 0.024) within the proximal nerve end. Within the distal nerve end, significant differences were observed between the autograft (2.00 ± 0.26), when compared to the hEC with saline (1.17 ± 0.31, *p* = 0.021), and the hEC with hMSC group (1.00 ± 0.10, *p* = 0.0071, Fig. 7A-B).

#### vWF

Expression of vWF was confirmed in all experimental groups with an increased level of vWF expression noted specifically in the hEC with hMSC at the distal end of conduit when compared to the autograft group. However, no significant differences were found between the hEC with hMSC, the hEC with saline and the autograft groups (Fig. 7C-D).

#### VEGF

The expression of VEGF was confirmed in all experimental groups. There was an increased level of VEGF expression at the proximal end of conduits, when compared to the distal ends. Furthermore, the hEC with hMSC group presented a higher value of VEGF expression than the autograft group at both ends of the nerve. Significant differences were detected between the hEC with hMSC at the proximal end of the conduit (2.67 ± 0.21) when compared to the hEC with hMSC at distal end (1.5 ± 0.22, *p* = 0.0156, Fig. 7E-F).

### Confirmation of Nerve Fibers Regeneration at 12-Weeks after Sciatic Nerve Gap Repair with hEC

Regeneration of the sciatic nerve in the autograft control as well as within the hEC experimental groups was evaluated using Toluidine staining and following assessments were made: myelin thickness, fiber diameter, percentage of myelinated fibers and axonal density.

#### Myelin Thickness

**Fig. 8:**
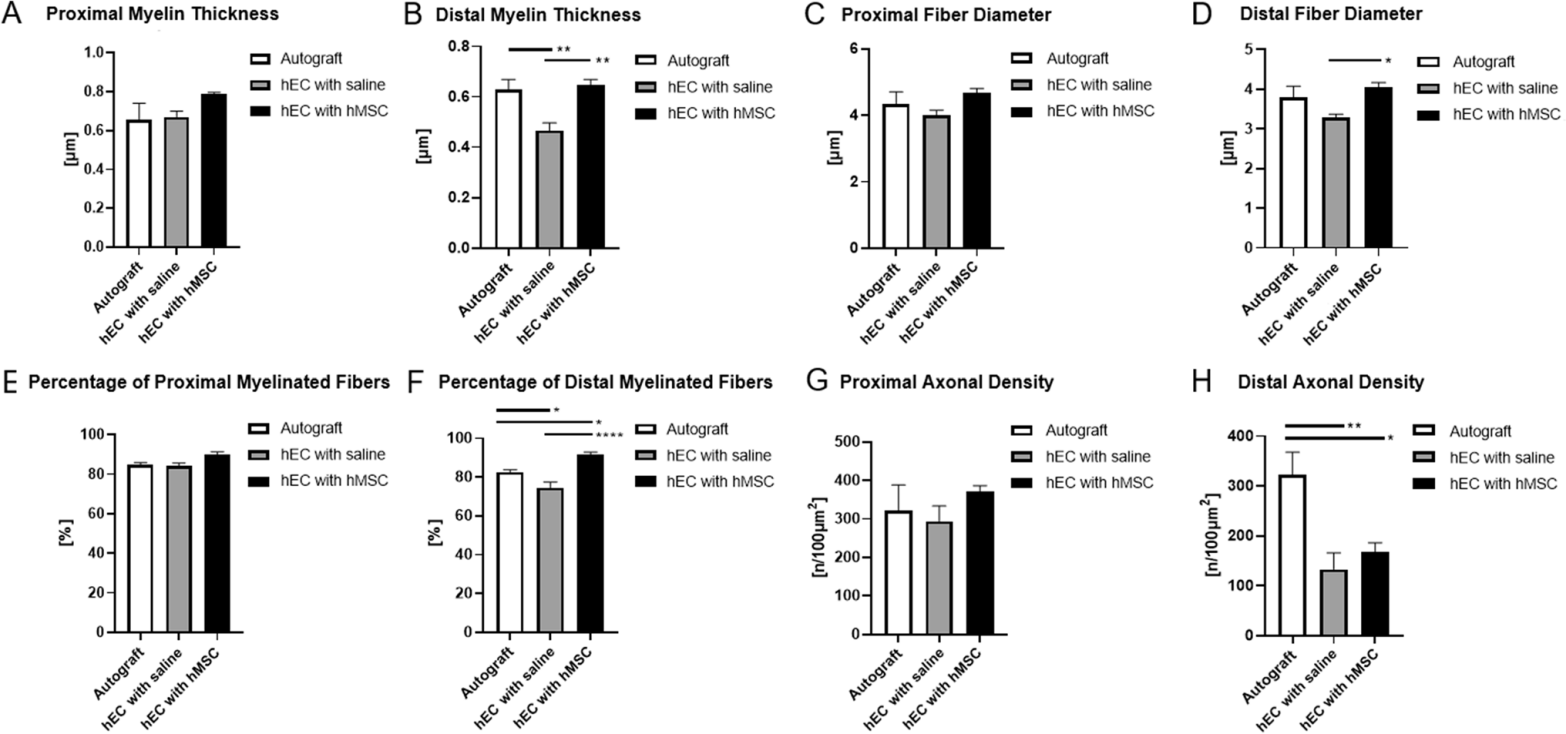
Histological assessment of the proximal and distal conduit ends at 12-weeks after nerve defect repair. **A** Proximal myelin thickness was increased in the hEC with hMSC group when compared to the hEC with saline and autograft groups. **B** Distal myelin thickness was significantly increased in the hEC with hMSC group when compared to the hEC with saline group and between the autograft and the hEC with saline group. **C** The largest proximal fiber diameter was observed in the hEC with hMSC treated group, followed by the autograft and the hEC with saline group. No significant results were observed. **D** Distal fiber diameter size was significantly greater in the hEC with hMSC when compared to the hEC with saline group. **E** The highest percentage of myelinated fibers at the proximal end was found in the hEC with hMSC group, followed by the autograft and the hEC with saline group. **F** The highest percentage of distal myelinated fibers was found in the hEC with hMSC group, followed by the autograft and the hEC with saline group. **G** The highest proximal axonal density was observed in the hEC with hMSC group, with no significant differences between the hEC with saline and the autograft groups. **H** The largest distal axonal density was observed in the autograft group, followed by the hEC with hMSC and the hEC with the saline group. The graphs represent mean values with SEM, statistical significance is marked with asterisks: *p < 0.05, **p < 0.01, ***p < 0.001, ****p < 0.0001

Proximal and distal myelin thickness was measured in the hEC with saline, the hEC with hMSC and in the autograft group at 12-weeks follow-up (Fig. 8A-B). Myelin thickness in distal nerve end was significantly increased in the autograft group compared to the hEC with saline (0.63 ± 0.04 vs. 0.47 ± 0.03; *p* = 0.0064), as well as between the hEC with saline and the hEC with hMSC (0.47 ± 0.03 vs. 0.65 ± 0.02; *p* = 0.003). No significant differences were detected between experimental groups within the proximal conduit end.

#### Fiber Diameter

At 12-weeks after nerve repair, a significant difference in distal fiber diameter size was observed in the hEC with hMSC group, when compared to the hEC with saline group (4.04 ± 0.01 vs. 3.29 ± 0.08; *p* = 0.0296, Fig. 8C-D). Assessment of proximal fiber diameter in the autograft, the hEC with hMSC and the hEC with saline groups revealed no significant differences.

#### Percentage of Myelinated Fibers

The percentage of myelinated fibers detected in the proximal and distal end of the conduit further supports the efficacy of the hEC with hMSC as the novel therapy for nerve regeneration. The collected data were calculated at 12-weeks post-surgery in the autograft control and both experimental groups. The average percentage of myelinated fibers at proximal end was higher in the hEC with hMSC group (89.7% ± 1.7%) compared to the autograft (84.7% ± 1.3%) or the hEC with saline (84.3% ± 1.3%) group but the differences were not significant. The distal ratio of myelinated fibers was significantly higher in the hEC with hMSC group (89.67% ± 1.71%) compare to the hEC with saline group (84.33% ± 1.31%, *p* < 0.0001) and the autograft group (84.67% ± 1.31%, *p* = 0.0109), confirming the supportive mechanism of hMSC on nerve regeneration. Significant difference was also detected between the autograft and the hEC with saline group (84.67% ± 1.31% vs. 84.33% ± 1.31%, *p* = 0.0226, Fig. 8E-F).

#### Axonal Density

At 12-weeks, the proximal axonal density was higher in the hEC with hMSC group (371/100 μm^2^ ± 16/100 μm^2^), when compared to the autograft (322/100 μm^2^ ± 66/100 μm^2^) and the hEC with saline (294/100 μm^2^ ± 40/100 μm^2^) groups. However, the differences were not significant. The values assessed for distal axonal density were significantly higher in the autograft group compared to the hEC with hMSC group (321.83 μm^2^ ± 45.87 μm^2^ vs. 167.33 μm^2^ ± 19.02 μm^2^, *p* = 0.0165) and the hEC with saline group (133.00 μm^2^ ± 33.17 μm^2^, *p* = 0.004). Moreover, distal axonal density was higher in the hEC with hMSC group (167/100 μm^2^ ± 47/100 μm^2^) when compared to the hEC with saline group (133/100 μm^2^ ± 81/100 μm^2^, Fig. 8G-H) confirming regenerative potential of hMSC during nerve regeneration.

## Discussion

Despite a considerable number of reports, the results of currently available therapeutic options for repair of peripheral nerves after injuries present several shortcomings and challenges for both the clinicians as well as the patients. Thus, novel therapeutic approaches are needed for the enhancement of peripheral nerve regeneration after trauma [33–36].

Numerous factors need to be carefully considered while optimizing the surgical approach in each patient. Factors that influence functional recovery after trauma include: the time since injury, patient’s age, type and level of injury, concomitant soft tissues damage, and vascular damage [5, 26]. Furthermore, the presence of soft and vascular tissue damage significantly slows the recovery process due to reduced circulation, development of adhesions and scar tissue formation in the affected area [26, 37].

Although direct nerve repair with use of the epineural microsurgical technique is a favored method of treatment, it is not applicable in the management of nerve gaps since the coaptation of the nerve ends under tension has adverse effect on nerve regeneration due to distortion of the microvascular flow. This, in consequence, leads to poor clinical outcomes. Terzis et al. noted that even marginal tension can adversely influence the functional results after nerve repair [6, 38].

In such cases, the application of bridging material between the two nerve stumps is needed to guide nerve regeneration from the proximal end to the distal end of the nerve. The lack of nerve guidance within the space between the two stumps may result in the misdirection of the regenerating axons, leading to neuroma formation. The isolation of regenerating nerve fibers is crucial to prevent painful neuroma formation [30]. In their study, Lundborg et al. have shown that a 10 mm nerve gap without guidance prevents regenerating fascicles from reaching the nerve’s distal end [39]. Multiple surgical strategies have been proposed to address these problems.

Autologous nerve grafts are viewed as the gold standard for bridging nerve gaps longer than 5 mm. They provide a favorable and stimulating scaffold by enhancing nerve regeneration and by the ability to supply Schwann cells, neurotrophic factors as well as endoneurial tube surface adhesion molecules. However, the autologous grafting method has its limitations, such as neuroma formation, tissue scar formation, and prolonged surgery. Limited availability, as well as differences in nerve diameter or insufficient length of the graft, may become other obstacles leading to poor nerve regeneration [38].

Nerve allografts from cadavers can be applied in segmental or complex nerve injuries where other surgical methods cannot be successfully applied. Unlimited supply, the avoidance of donor site morbidities such as neuroma formation, scarring and sensory loss, and ability to bridge the nerve gap have made the allograft a readily accessible alternative to other methods. However, the use of allografts requires systemic immunosuppression [26]. Due to the side effects of immunosuppressive therapy and access to other alternative methods of nerve repair, such as nerve transfers or conduits, allografts option should be reserved for patients with complex, irreparable nerve damage resulting in the essential functional deficits [40, 41].

One of the strategies includes nerve transfer techniques [42]. In the last 20 years, multiple authors published impressive results employing this technique. Nerve transfer technique is commonly used in brachial plexus or other proximal injuries, where long distance from target motor endplates occurs. Advantages of nerve transfer include avoidance of autograft and associated donor site morbidity, also providing earlier reinnervation. On the other hand, this technique leads to the loss of function from the donor nerve site and is limited to isolated nerve injuries. Moreover, the donor’s muscle is no longer available for muscle transfer [43, 44].

Other alternative methods for nerve gap repair include biological and artificial conduits. Guidance tubes provide protection for the growing axons from the surrounding tissues, assist in directing axons toward the distal nerve stump, restrict inflammation and fibrosis as well as hinder the formation of a neuroma. A considerable number of natural conduits has been investigated so far. These include skeletal muscle tissue, tendons, and vessels as a potential alternative to the autografts. Biological materials have lower toxicity and increased compatibility when compared with synthetic materials; however, they may not be suitable for longer nerve gaps. Autologous venous nerve conduits showed similar results in returning nerve conduction velocity when compared to the conventional nerve grafts. However, vein grafts tend to collapse, resulting in disturbance of the recovery process. Thus, muscle tissue, as well as bone marrow stromal cells, were placed into the vein grafts to investigate whether this would prevent vein grafts from collapsing. Several investigators reported some promising results, although the most satisfactory nerve recovery was observed after nerve autograft repair [45–47]. Our Laboratory assessed isogenic venous graft supported with bone marrow stromal cells (BMSC) as a natural conduit for bridging a 20 mm nerve gap. Our results confirmed that injection of BMSC into the vein grafts improved nerve regeneration and prevented vein graft from collapsing [48].

Synthetic nerve guide conduits, explored as a possible alternative method for peripheral nerve gap repair, are created from biodegradable or non-degradable materials. Implementation of non-human nerve conduits in peripheral nerve gap management reduces damage to the donor’s nerves during nerve reconstruction [49]. Silicone conduits are constructed from non-degradable material. Their neuroregenerative potential has been studied for many years and has been shown to promote nerve recovery. However, the widespread application of the silicon conduits in the clinical setting has been hindered by the evidence of fibrous tissue formation and chronic inflammatory response due to the foreign body creation. This may lead to formation of adhesions, nerve scarring and constriction, which may require additional surgical procedure for the silicone tube removal [50, 51].

Due to the problems encountered with the non-degradable conduits, researchers have focused on creating conduits from biodegradable materials such as collagen, polyglycolic acid, chitosan, polyester, and copolyesters, all have been studied as the alternative methods for bridging nerve gaps after trauma [49, 52, 53].

Due to the limitations of the current surgical methods for repair of long nerve defects, novel strategies are required to improve nerve regeneration. Epineurium, as a natural component of the nerve, provides favorable microenvironment conditions that promote Schwann cells attachment and, therefore, supports axonal growth. Its neurotrophic and angiogenic properties make epineurium a desirable material for the creation of a conduit. In our previous studies, we have proven that epineural sheath is supportive for nerve regeneration and eliminates complications related to the autograft harvesting procedure including neuroma formation, tissue scarring, prolonged surgery, as well as limited nerve supply [15, 30, 31]. Furthermore, Siemionow et al. assessed the application of epineural sheath conduits for the repair of long nerve gaps. In that study, Siemionow et al. confirmed the feasibility of the application of epineural sheath conduits for the restoration of a 6 cm long nerve defect in sheep - the large animal model [14].

It is well established that the local microenvironment is essential during the process of nerve regeneration. The regenerative capacity of Schwann cells (SC) within nerve tubes promotes proliferation, myelination, and regeneration of sprouting axons [54]. However, the volume of SCs is confined, and their doubling time is slow; thus, there is a need for selecting highly proliferative cells with functional properties of Schwann cells [55].

In this study, we combined the use of human epineural conduit with the human mesenchymal stem cells in order to enhance sciatic nerve regeneration after injury. Adult bone-marrow-derived mesenchymal stem cells (MSC) have the ability to extensively differentiate into multiple cell lineages. These cells are known for their regenerative potential as they induce damaged cells removal and replacement, generation of growth factors and immunomodulatory properties. Thus, MSC are used in the treatment of different diseases. MSC stimulates neurogenesis by the synthesis of factors that influence angiogenesis, immune response, neuronal cell survival and proliferation. MSC beneficial therapeutic effect has been proven in neurological disorders such as stroke and traumatic brain injury. Among secreted neuro-regulatory proteins, the most valuable are: BDNF and (beta)-NGF, CNGF and IGF. BDNF supports neurite outgrowth by activation of Schwann Cells through the activation of the JAK/STAT pathway [20]. IGF-1 supports neuronal survival, promotes neurite growth and differentiation, as well as enhances functional recovery [21, 56]. Other investigators suggested using adipose-derived stem cells (ASC) as a source of growth, neurotrophic and angiogenic molecules. They reported comparable findings to our study, however in short-term evaluation. In contrast, our study evaluated regenerative properties of MSC at 12-weeks after nerve repair with hEC supported with hMSC [57, 58].

In our study the motor and sensory functional recovery was assessed by standard toe-spread and pinprick tests up to 12-weeks after nerve repair. As expected at the 12-weeks study endpoint toe-spread test revealed the best recovery in the autograft group. There was no significant difference between the autograft group and the hEC supported with hMSC group, which confirms comparable regenerative potential of both methods of nerve repair after trauma. In the short-term observation (6-weeks) epineural conduit promoted faster motor recovery than the autograft control as shown by toe-spread score. At the 12-weeks observation there was no statistical difference in pinprick test between the autograft and the hEC with hMSC group. The differences were significant between the autograft and the hEC with saline groups. Gastrocnemius Muscle Index (GMI), which assessed muscle denervation atrophy, reached the highest value in the autograft group at the end of the study. Muscle fiber area ratio revealed the most favorable results in the autograft group, closely followed by hEC supported with hMSC and hEC with saline group, respectively. Histomorphometric evaluation with toluidine blue staining revealed significant differences in myelin thickness, fiber diameter, and percentage of myelinated fibers within distal nerve stump between hEC with saline group when compared to hEC with hMSC group, which confirmed the efficacy of human mesenchymal stem cells in the enhancement of nerve regeneration. Fiber diameter, as well as myelin thickness, were comparable between the autograft and hEC with hMSC group, further indicating that human epineural conduit supported with hMSC may be considered as an alternative method to the autograft repair. Similar findings were reported by other investigators, where the influence of collagen guidance tubes supported with bone marrow-derived cells (BMDCs) on sciatic nerve regeneration in mice was investigated. They reported significant differences in the number of myelinated fibers, nerve fiber area and myelin sheath area in the experimental group supported with BMDCs when compared to the control group [59].

In the current study immunofluorescent staining revealed the presence of PKH26 (the membrane dye labeling hMSC) only in the hEC filled with hMSC group when compared to the saline-injected conduits. These findings confirm that at 12-weeks after injection the hMSC were still present within the conduits. Our previous studies assessing enhancement of nerve defect regeneration with epineural tubes supported with bone marrow stromal cells (BMSC) also revealed the presence of BMSC at 12-weeks after injection into a conduit in the rat model [15]. Immunostaining has not revealed significant differences in expression of proneurogenic factors: Laminin B, S-100 and GFAP between autograft and hEC with hMSC group, which confirms similar dynamics of the nerve regeneration in both the autograft group and the hEC supported with hMSC group. NGF expression reached the highest values in the autograft group, which were significant when compared to the hEC with hMSC as well as the hEC with saline groups. A trend for increased expression of neurogenic factors was observed within the autograft and the hEC supported with hMSC when compared to hEC with saline or no repair group. Expression of GFAP, Laminin B and S-100 was higher in the hEC with hMSC when compared to the hEC with saline group within the proximal and distal end of the conduit further confirming neuroprotective properties of hMSC. Good vascularization after nerve injury is essential for nerve regeneration, as it maintains proper blood supply required for long-term tissue regeneration. In our study, the level of VEGF expression was highest in the hEC with hMSC group, however this difference did not reach significance. Petrova et al. studied rat peripheral nerve angiogenesis after injury and MSC delivery. They observed increased number and density of blood vessels in the regenerating nerve after MSC administration during the nerve injury in the experimental group [60]. Influence of MSC on nerve regeneration was also investigated after stem cells delivery to artificial nerve conduit. The authors have chosen human umbilical cord mesenchymal stem cells (hUC-MSCs) as a source of neurogenic and angiogenic factors supporting nerve regeneration, due to their accessibility, self-renewal capacity, hypo-immunogenic and non-tumorigenic properties. In the study, a 3.5 cm defect of the sciatic nerve in dogs was bridged with a longitudinally oriented collagen conduit (LOCC) supported with hUC-MSCs. LOCC combined with hUC-MSCs resulted in better functional recovery when compared to the LOCC alone group, however improvement was inferior to the autologous nerve graft group [61]. In our study, the hEC supported with hMSC group confirmed comparable regenerative capacity to the autograft group in terms of functional recovery, GMI and histomorphometric parameters.

In conclusion, in this study, we assessed the efficacy of the hEC conduit supported with hMSC in the enhancement of nerve regeneration following nerve injury. We confirmed successful application of allogeneic human epineural conduit in the repair of peripheral nerve defects assessed in the nude rat experimental model up to 12-weeks after nerve gap repair. Considering abundant epineurium supply, accessibility, and the lack of immune response, the epineural conduit, supported with hMSC, introduces a new promising alternative to the autograft technique, which represents the current gold standard of peripheral nerve gap repair. Although more research is warranted to define the storage and preservation protocols of the conduits and the optimization of MSC harvesting and propagation, we believe that human epineural conduit can be used in the future as the “off-the-shelf” product allowing for fast and straightforward clinical application for reconstruction of peripheral nerve defects after trauma.

## Data Availability

All data generated or analyzed during this study are included in this published article author.
